# Characteristic analysis of adverse reactions of five anti-TNFɑ agents: a descriptive analysis from WHO-VigiAccess

**DOI:** 10.3389/fphar.2023.1169327

**Published:** 2023-07-24

**Authors:** Mingming Li, Ruxu You, Yuyong Su, Hongbo Zhou, Shiwei Gong

**Affiliations:** ^1^ Department of Pharmacy, Union Hospital, Tongji Medical College, Huazhong University of Science and Technology, Wuhan, China; ^2^ School of Pharmacy, Tongji Medical College, Huazhong University of Science and Technology, Wuhan, China

**Keywords:** adverse drug reaction, TNFα inhibitors, pharmacovigilance, spontaneous reporting, VigiAccess of the WHO

## Abstract

**Introduction:** Tumor necrosis factor (TNF) inhibitors (adalimumab, infliximab, etanercept, golimumab, and certolizumab pegol) have revolutionized the treatment of severe immune-mediated inflammatory diseases, including rheumatoid arthritis, Crohn’s disease, psoriatic arthritis, ankylosing spondylitis, and ulcerative colitis. This study assessed adverse drug reactions (ADRs) after the use of TNFα inhibitors in VigiAccess of the World Health Organization (WHO) and compared the adverse reaction characteristics of five inhibitors to select the drug with the least risk for individualized patient use.

**Methods:** The study was a retrospective descriptive analysis method in design. We sorted out five marketed anti-TNFα drugs, and their ADR reports were obtained from WHO-VigiAccess. Data collection included data on the age groups, sex, and regions of patients worldwide covered by ADR reports, as well as data on disease systems and symptoms caused by ADRs recorded in annual ADR reports and reports received by the WHO. By calculating the proportion of adverse reactions reported for each drug, we compared the similarities and differences in adverse reactions for the five drugs.

**Results:** Overall, 1,403,273 adverse events (AEs) related to the five anti-TNFα agents had been reported in VigiAccess at the time of the search. The results show that the 10 most commonly reported AE manifestations were rash, arthralgia, rheumatoid arthritis, headache, pneumonia, psoriasis, nausea, diarrhea, pruritus, and dyspnea. The top five commonly reported AE types of anti-TNFα drugs were as follows: infections and infestations (184,909, 23.0%), musculoskeletal and connective tissue disorders (704,657, 28.6%), gastrointestinal disorders (122,373, 15.3%), skin and subcutaneous tissue disorders (108,259, 13.5%), and nervous system disorders (88,498, 11.0%). The preferred terms of myelosuppression and acromegaly were obvious in golimumab. Infliximab showed a significantly higher ADR report ratio in the infusion-related reaction compared to the other four inhibitors. The rate of ADR reports for lower respiratory tract infection and other infections was the highest for golimumab.

**Conclusion:** No causal associations could be established between the TNFα inhibitors and the ADRs. Current comparative observational studies of these inhibitors revealed common and specific adverse reactions in the ADR reports of the WHO received for these drugs. Clinicians should improve the rational use of these high-priced drugs according to the characteristics of ADRs.

## Introduction

Tumor necrosis factor (TNF)α is a proinflammatory, multifunctional cytokine that is synthesized by various cells, including activated monocytes, macrophages, and T cells (Darrigade et al., 2017). TNFα has been shown to play an essential role in the pathogenesis of autoimmune diseases such as rheumatoid arthritis (RA), psoriatic arthritis (PSA), and ankylosing spondylitis (AS). Therefore, drugs targeting TNF have been developed to neutralize the effects of these pro-inflammatory cytokines (Jang et al., 2021). Anti-TNFα drugs are usually well-tolerated; however, there have been reports of many potentially serious adverse effects. Long-term use of anti-TNFα agents has been associated with the risk of serious infections, malignancies, skin and soft tissue infections, and tuberculosis (Kroesen et al., 2003; Shivaji et al., 2019). A meta-analysis showed that treatment with anti-TNFα agents increased the risk of serious infections (OR: 1.72, 95% CI: 1.56–1.90, *p* < 0.00001) and an increase in cancer risk (OR: 1.36, 95% CI: 1.20–1.53, *p* < 0.00001), whereas the risk of tuberculosis was not significantly different (Li et al., 2021). Although there was no consensus on the risk of infection associated with anti-TNFα treatments in published clinical trials, post-marketing surveillance and retrospective studies have shown an increased risk of tuberculosis and other granulomatous infections (Wallis et al., 2004; Godfrey and Friedman, 2019; Athimni et al., 2022; Doughty et al., 2023). A meta-analysis carried out in 2006 showed an increase in the risk of malignancies and serious infections in patients treated with infliximab and adalimumab, where a higher dose was associated with increased cancer risk (Galloway et al., 2011). Despite the rigor of pre-marketing drug trials, the safety of medicines is not completely understood from pre-authorization clinical trial data as these trials are conducted in controlled settings different from settings of real-world use (Gagliardi et al., 2022). In particular, biological agents such as TNFα inhibitors, which have been on the market for a long time, have a large population base and a wide range of use, so safety research based on a large number of real-world data is more appropriate and valuable. Therefore, adverse drug reactions (ADRs) associated with the TNFα inhibitors need to be further characterized from spontaneous reports in pharmacovigilance databases. More significantly, there are no studies to compare the similarities and differences of ADRs produced by these drugs.

Despite the intrinsic limitations, spontaneous reporting systems represent a valuable source to obtain real-world data about the safety profile of drugs and vaccines, compare therapeutic options, and gain insight into the potential mechanisms of ADRs (Hazell and Shakir, 2006). Spontaneous reporting systems have been the backbone of pharmacovigilance since their introduction in the 1960s. The main aim of spontaneous reporting is the early detection of previously unrecognized ADRs. In addition, spontaneous reporting can also be useful for obtaining information on new aspects of known associations between drugs and ADRs (Srba et al., 2012). The Uppsala Monitoring Center (UMC), on behalf of the World Health Organization (WHO)’s Programme for International Drug Monitoring (PIDM), brings together safety data from all corners of the world. As of the end of 2018, the UMC had received and stored over 20 million ADR reports from more than 170 countries in VigiBase, which is a worldwide voluntary reporting program. Since 2015, data stored in VigiBase can be freely accessed by the public via VigiAccess (Watson et al., 2018; Habarugira and Figueras, 2021). The VigiAccess database supports searching by the trade name of the drug, but the database will identify the active ingredient it contains and display the results of its ADR reports according to the active ingredient. This study searched for five biological TNFα blockers approved by the Food and Drug Administration (FDA): adalimumab, infliximab, etanercept, golimumab, and certolizumab pegol.

The five TNFα inhibitors showed similar efficacy profiles, although recent data show that adalimumab seems to be most effective in geriatric patients, while etanercept is associated with a lower risk of developing *tuberculosis* (Bonek et al., 2021). Therefore, clinicians are often required to tailor treatment decisions based on the risk of adverse events for the individual patient. To compare the differences in the occurrence of these five anti-TNFα-related adverse reactions, we conducted a descriptive study of spontaneous reported adverse reactions in the VigiAccess database and compared the reported rates of adverse reactions caused by these five drugs.

## Materials and methods

### Drug sample

Table 1 shows the five anti-TNFα agents that we have studied that are available for clinical use. Humanized anti-TNF-α mAbs include Humira^®^ (adalimumab), Simponi^®^ (golimumab), and Cimzia^®^ (certolizumab pegol). Remicade^®^ (infliximab) was the first chimeric anti-TNF-α mAb for the treatment of Crohn’s disease. The use of Remicade was then extended to other therapeutic areas, including RA (in combination with methotrexate), ankylosing spondylitis, psoriatic arthritis, ulcerative colitis, pediatric Crohn’s disease, plaque psoriasis, and pediatric ulcerative colitis. The first Ab fusion protein, Enbrel^®^ (etanercept), was approved by the FDA for clinical use in patients with RA in 1998. It comprises the Fc region of Ab conjugated with TNF-receptor 2 (TNFR2). The approval of etanercept opened up the way for the development of several recombinant protein methodologies based on the fusion of various proteins to different antibody regions, including single-chain variable fragments (scFvs), heavy-chain Abs (hcAbs), single-chain Abs (scAbs), and antigen-binding fragments (Fabs) (Leone et al., 2023). It can be seen in Table 1 that their structures are not completely the same, and the sources of synthesis are also different. The earliest launch time of these five drugs is more than 10 years, and they are currently among the world’s best-selling drugs, and all of them are marketed in China.

**TABLE 1:** General information of five anti-TNFɑ inhibitors.

Drug name and brand name	Structure	Main conditions	First marketing time	Biosimilars
Adalimumab–Humira	Human monoclonal antibodies	Rheumatoid arthritis and ankylosing spondylitis	2002	Amgevita, Cyltezo, Imraldi, Solymbic, Yusimry, Halimatoz, Hefiya, Hyrimoz, Idacio, Kromeya, Hadlima, Abrilada, and Hulio
Infliximab–Remicade	Chimeric monoclonal antibody	Rheumatoid arthritis, ankylosing spondylitis, and ulcerative colitis	1998	Remsima, Inflectra, Flixabi, Renflexis, Ixifi, Zessly, and Avsola
Etanercept–Enbrel	Receptor construct	Psoriasis, rheumatoid arthritis, and ankylosing spondylitis	1998	Erelzi, Benapali, and Eticovo
Golimumab–Simponi	Human monoclonal antibodies	Rheumatoid arthritis, ankylosing spondylitis, and ulcerative colitis	2009	-
Certolizumab pegol–Cimzia	Fab’ fragment of humanized monoclonal antibody	Rheumatoid arthritis	2008	-

By December 2022, there were thirteen adalimumab biosimilars, seven infliximab biosimilars, and three etanercept biosimilars (Table 1). There was no increase in adverse events (AEs) in patients treated with adalimumab biosimilars and concomitant methotrexate (MTX) therapy compared to Humira and MTX therapy in patients with RA (Cohen et al., 2017; Fleischmann et al., 2018). Studies have shown that there are no differences in safety, immunogenicity, and pharmacokinetics between infliximab and infliximab biosimilars (Subedi et al., 2019). Only proven similar efficacy and safety in short- and long-term studies allow switching between originator and biosimilar products. The current time available for comparative research is limited (Agca et al., 2017; Kameda et al., 2020).

### Data sources

WHO-VigiAccess was searched on 2 January 2022 for all reported adverse events following the introduction of anti-TNFα agents. The login URL is https://www.vigiaccess.org. All study drugs were identified by the generic name. Data were captured among age groups, sex, report year, and continents of the world by WHO-VigiAccess. Descriptive data were calculated using Excel 2019 version.

WHO-VigiAccess is a free-access portal to the PIDM database allowing retrieval of medicinal products’ safety reports received by the UMC. The definition relied on system organ class (SOC) and preferred terms (PTs) by the Medical Dictionary for Regulatory Activities (MedDRA). Thus, records on each anti-TNFα drug were retrieved, and all individual AEs based on MedDRA SOC and PT levels recorded were identified to describe the spectrum of toxicities. Reporting terms used in MedDRA were derived from several dictionaries, including the WHO Adverse Reaction Terminology (WHO-ART), among others (Sultana et al., 2020). A total of 27 items were classified by SOC, of which 20 items directly related to disease symptoms were selected for analysis. In the present study, we focused on the PTs, the level used in the VigiBase database publicly accessible information via WHO-VigiAccess.

To study the outcomes of detected safety signals, we grouped them using outcome code to produce the three severe categories: death, hospitalization, and major events comprising life-threatening events, disability, and congenital anomaly.

### Statistical analysis

The study followed a retrospective quantitative study design. Excel descriptive analysis was used to analyze the characteristics of the victims of adverse reactions to the five drugs. The ADR symptom number of each drug divided by the total number of ADR reports was defined as the ADR report rate of the drug. Common ADRs of each drug refer to the symptoms of the top 20 ADR report rate. The rate of reported ADR symptoms for each drug was calculated, and a descriptive comparative analysis was performed. Frequencies and percentages were used to categorize descriptive variables.

## Results

### Description of the studied cases

The earliest reports of adalimumab, infliximab, etanercept, golimumab, and certolizumab pegol adverse reactions were received in the WHO-VigiAccess database in 2001, 1999, 1999, 2008, and 2003, respectively. By 2021, the WHO had received a total of 591,705, 172,961, 542,647, 38,629, and 57,331 ADR reports for these five drugs, with a total of 1,403,273 reports. The numbers of adverse events covered in these ADR reports were 840,417 cases of adalimumab, 280,811 cases of infliximab, 654,269 cases of etanercept, 4,895 cases of golimumab, and 78,388 cases of certolizumab pegol. Among the 1,403,273 reports related to the five anti-TNFα agents shown in Table 2, except for 73,803 cases in which the sex was unknown, the number of women (916,873) who had ADRs was significantly greater than that of men (412,597), and the female–male ratio was 2.22:1 with a big discrepancy. Excluding the unknown age reports, most of the age groups with the highest reported rates are between 45 and 64 years. Most of the AEs were reported from the Americas (77.94%). Table 2 also lists the reporting years for each of the studied drugs.

**TABLE 2:** Characteristics of ADR reports of five anti-TNFα drugs.

	Adalimumab	Infliximab	Etanercept	Golimumab	Certolizumab pegol
Number of ADR reports	591,705	172,961	542,647	38,629	57,331
Female	379,621 (64.2%)	94,131 (54.4%)	376,547 (69.4%)	25,123 (65.0%)	41,451 (72.3%)
Male	191,141 (32.3%)	62,859 (36.3%)	134,050 (24.7%)	11,425 (29.6%)	13,122 (22.9%)
Unknown	20,943 (3.5%)	15,971 (9.2%)	32,050 (5.9%)	2,081 (5.4%)	2,758 (4.8%)
<18	10,908 (1.8%)	7,587 (4.4%)	11,824 (2.2%)	220 (0.6%)	406 (0.7%)
18–44	107,447 (18.2%)	45,770 (26.5%)	73,786 (13.6%)	7,147 (18.5%)	10,948 (19.1%)
45–64	149,288 (25.2%)	42,258 (24.4%)	198,184 (36.5%)	12,867 (33.3%)	12,863 (22.4%)
65–74	47,616 (8.5%)	14,022 (8.1%)	67,265 (12.4%)	4,538 (11.8%)	4,326 (7.6%)
>75	18,482 (3.1%)	5,771 (3.3%)	27,566 (5.1%)	1,904 (4.9%)	1,735 (3.0%)
Unknown	257,964 (43.6%)	57,553 (33.3%)	164,022 (30.2%)	11,953 (30.9%)	27,053 (47.2%)
Africa	1,369 (0.2%)	760 (0.4%)	725 (0.1%)	151 (0.4%)	9 (˗)
Americas	445,918 (75.4%)	118,881 (68.7%)	467,392 (86.1%)	21,974 (56.9%)	39,517 (68.9%)
Asia	9,484 (1.6%)	9,962 (5.8%)	5,582 (5.4%)	2,094 (5.4%)	1,241 (2.2%)
Europe	131,479 (22.2%)	40,717 (23.5%)	67,945 (35.7%)	13,804 (35.7%)	16,274 (28.4%)
Oceania	3,455 (0.6%)	2,641 (1.5%)	1,003 (0.2%)	606 (1.6%)	290 (0.5%)
Before 2010	57,603 (9.7%)	35,133 (20.3%)	85,186 (15.7%)	184 (0.5%)	3,313 (5.8%)
2011	21,489 (3.6%)	10,656 (6.2%)	26,142 (4.8%)	537 (1.4%)	5,502 (9.6%)
2012	27,922 (4.7%)	12,051 (7.0%)	35,429 (6.5%)	1,260 (3.3%)	1,123 (2.0%)
2013	7,695 (1.3%)	8,453 (4.9%)	25,420 (4.7%)	1,211 (3.2%)	873 (1.5%)
2014	73,612 (12.4%)	7,371 (4.3%)	55,061 (10.2%)	1,204 (3.1%)	1,696 (3.0%)
2015	49,219 (8.3%)	12,570 (7.3%)	68,668 (12.7%)	4,390 (3.1%)	3,180 (5.6%)
2016	84,741 (14.3%)	25,867 (15.0%)	44,654 (8.2%)	4,654 (11.4%)	5,879 (10.3%)
2017	37,432 (6.3%)	12,761 (7.4%)	59,387 (10.9%)	4,243 (12.1%)	3,525 (6.2%)
2018	61,948 (10.5%)	17,325 (10.0%)	60,154 (11.1%)	6,591 (11.0%)	6,506 (11.4%)
2019	66,396 (11.2%)	13,265 (7.7%)	45,022 (8.3%)	6,623 (17.1%)	6,017 (10.5%)
2020	53,381 (9.0%)	7,158 (4.1%)	18,163 (3.4%)	3,727 (9.7%)	12,012 (21.0%)
2021	48,669 (8.2%)	9,972 (5.8%)	18,168 (3.4%)	3,831 (9.9%)	7,505 (13.1%)

### Distribution of 20 SOCs of five anti-TNFα drugs

Table 3 shows the report rates of 20 SOCs of five anti-TNFα drugs. Adalimumab-related nervous system disorders and skin and subcutaneous tissue disorders were significantly higher than the disorders of the other four TNFα inhibitors. The rates of ADR reports of infliximab-related gastrointestinal disorders, cardiac disorders, benign, malignant, and unspecified neoplasms, vascular disorders, and respiratory, thoracic, and mediastinal disorders were significantly higher than those of the other four TNFα inhibitors. Higher rates of ADRs were reported for musculoskeletal and connective tissue disorders in etanercept, as well as infections and infestations in golimumab.

**TABLE 3:** ADR number and report rate of 20 SOCs of five anti-TNFɑ drugs.

System organ classes	Adalimumab (N = 591705)	Infliximab (N = 172961)	Etanercept (N = 542647)	Golimumab (N = 38629)	Certolizumab pegol (N = 57331)
Blood and lymphatic system disorders	9,436 (1.59%)	3,918 (2.26%)	7,403 (1.36%)	566 (1.47%)	739 (1.29%)
Cardiac disorders	14,795 (2.50%)	7,695 (4.45%)	8,920 (1.64%)	831 (2.15%)	1,157 (2.02%)
Congenital, familial, and genetic disorders	1,155 (0.19%)	476 (0.28%)	901 (0.17%)	60 (0.16%)	143 (0.25%)
Ear and labyrinth disorders	5,658 (0.96%)	1,074 (0.62%)	5,239 (0.97%)	252 (0.65%)	393 (0.69%)
Endocrine disorders	1860 (0.31%)	552 (0.32%)	1,182 (0.22%)	103 (0.27%)	116 (0.20%)
Eye disorders	16,608 (2.81%)	4,192 (2.42%)	14,422 (2.66%)	817 (2.11%)	1,228 (2.14%)
Gastrointestinal disorders	95,600 (16.16%)	35,274 (20.39%)	42,005 (7.74%)	3,603 (9.32%)	9,219 (16.08%)
Hepatobiliary disorders	6,616 (1.12%)	3,183 (1.84%)	3,602 (0.64%)	365 (0.94%)	525 (0.92%)
Immune system disorders	13,119 (2.22%)	10,015 (5.79%)	14,347 (2.64%)	857 (2.22%)	1,616 (2.82%)
Infections and infestations	127,859 (21.61%)	42,750 (24.72%)	111,993 (20.64%)	10,796 (27.95%)	15,192 (26.50%)
Metabolism and nutrition disorders	15,241 (2.58%)	4,116 (2.38%)	7,684 (1.42%)	541 (1.40%)	1,025 (1.79%)
Musculoskeletal and connective tissue disorders	100,743 (17.02%)	21,237 (12.28%)	106,799 (19.68%)	5,221 (13.52%)	8,222 (14.34%)
Benign, malignant, and unspecified neoplasms (including cysts and polyps)	22,364 (3.78%)	13,143 (7.60%)	16,972 (3.13%)	1,658 (4.29%)	1,773 (3.09%)
Nervous system disorders	68,527 (11.58%)	17,238 (9.97%)	53,879 (9.93%)	2,966 (7.68%)	5,244 (9.15%)
Psychiatric disorders	26,879 (4.54%)	4,591 (2.65%)	19,026 (3.51%)	1,018 (2.64%)	1,866 (3.25%)
Renal and urinary disorders	11,579 (1.96%)	3,900 (2.25%)	6,615 (1.22%)	582 (1.51%)	958 (1.67%)
Reproductive system and breast disorders	7,352 (1.24%)	1,616 (0.93%)	4,108 (0.76%)	272 (0.70%)	545 (0.95%)
Respiratory, thoracic, and mediastinal disorders	48,280 (8.16%)	19,083 (11.03%)	42,051 (7.75%)	2,160 (5.59%)	3,928 (6.85%)
Skin and subcutaneous tissue disorders	84,545 (14.29%)	23,545 (13.61%)	69,874 (12.88%)	3,558 (9.21%)	7,621 (13.29%)
Vascular disorders	17,960 (3.04%)	11,837 (6.84%)	10,631 (1.96%)	964 (2.50%)	1,381 (2.41%)

The top five commonly reported AE types of anti-TNFα drugs were as follows: infections and infestations (184,909, 23.0%), musculoskeletal and connective tissue disorders (704,657, 28.6%), gastrointestinal disorders (122,373, 15.3%), skin and subcutaneous tissue disorders (108,259, 13.5%), and nervous system disorders (88,498, 11.0%). The rate of ADRs reported more than 10% in the SOC, and there were four in adalimumab, five in infliximab, three in etanercept, two in golimumab, and four in certolizumab pegol.

### Most common ADRs of five anti-TNFα drugs

The 20 most commonly reported ADRs of the five drugs are presented in Table 4, and the manifestations listed were preferred terms from within the SOC. The common ADRs of all five TNFα inhibitors were rash, arthralgia, rheumatoid arthritis, headache, pneumonia, psoriasis, nausea, diarrhea, and pruritus. Infliximab showed a significantly higher ADR report rate in the infusion-related reaction compared to the other four inhibitors. The rate of ADR reports of lower respiratory tract infection and other infections in golimumab ranks at the top. Most of the AEs in the top 20 commonly reported were minor events that are self-limiting. However, there were some noteworthy events, and these were Crohn’s disease, rheumatoid arthritis, and all infections (pneumonia, urinary tract infection, and nasopharyngitis).

**TABLE 4:** Top 20 ADRs of anti-TNFα drugs.

Adalimumab (N = 591705)		Infliximab (N = 172961)		Etanercept (N = 542647)		Golimumab (N = 38629)		Certolizumab pegol (N = 57331)	
ADR	Report rate %	ADR	Report rate %	ADR	Report rate %	ADR	Report rate %	ADR	Report rate %
Arthralgia	5.07	Infusion-related reaction	6.05	Arthralgia	5.52	Lower respiratory tract infection	3.44	Rash	3.57
Headache	3.56	Dyspnea	4.71	Rheumatoid arthritis	4.11	Rheumatoid arthritis	3.17	Arthralgia	3.44
Psoriasis	3.33	Crohn’s disease	4.07	Psoriasis	3.96	Pneumonia	3.09	Rheumatoid arthritis	3.28
Nasopharyngitis	3.12	Pneumonia	3.75	Pain in extremity	3.53	Arthralgia	2.71	Crohn’s disease	3.04
Nausea	3.01	Arthralgia	3.09	Headache	3.36	Nasopharyngitis	2.54	Nausea	2.90
Diarrhea	2.76	Abdominal pain	2.87	Nasopharyngitis	2.98	Infection	2.35	Headache	2.87
Rash	2.58	Nausea	2.85	Sinusitis	2.26	Rash	1.99	Diarrhea	2.84
Crohn’s disease	2.58	Rash	2.51	Nausea	2.14	Headache	1.82	Nasopharyngitis	2.80
Pain in extremity	2.56	Pruritus	2.32	Cough	2.10	Urinary tract infection	1.61	Infection	2.71
Rheumatoid arthritis	2.05	Headache	2.23	Joint swelling	1.88	Nausea	1.58	Pneumonia	2.50
Abdominal pain	1.90	Diarrhea	2.14	Pneumonia	1.85	Influenza	1.44	Psoriasis	2.15
Pneumonia	1.87	Flushing	2.05	Rash	1.76	Colitis ulcerative	1.41	Abdominal pain	2.10
Pruritus	1.86	Vomiting	1.97	Lower respiratory tract infection	1.62	Psoriasis	1.40	Urinary tract infection	1.96
Cough	1.79	Hypersensitivity	1.90	Pruritus	1.62	Diarrhea	1.34	Pruritus	1.86
Infection	1.71	Urticaria	1.89	Infection	1.58	Alopecia	1.18	Sinusitis	1.72
Dizziness	1.71	Colitis ulcerative	1.80	Back pain	1.48	Pain in extremity	1.15	Lower respiratory tract infection	1.68
Dyspnea	1.56	Erythema	1.73	Diarrhea	1.39	Pruritus	1.14	Dizziness	1.61
Vomiting	1.56	Psoriasis	1.58	Dizziness	1.38	Dyspnea	1.05	Influenza	1.44
Back pain	1.55	Dizziness	1.34	Musculoskeletal stiffness	1.35	Dizziness	1.02	Dyspnea	1.42
Sinusitis	1.51	Cough	1.24	Influenza	1.23	Joint swelling	0.99	Pain in extremity	1.41

### Serious AEs of five anti-TNFɑ drugs

**FIGURE 1:**
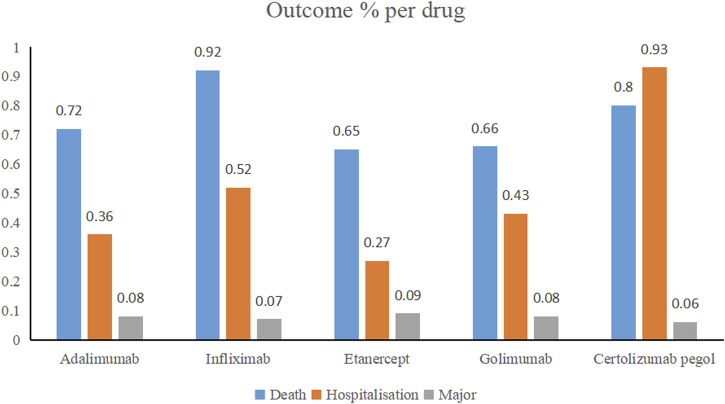
Outcomes for serious adverse events associated with anti-TNFα drugs at the level of preferred terms (major events comprising life-threatening events, disability, and congenital anomaly).

Through WHO-VigiAccess, we can also find major adverse events of anti-TNFα drugs, including life-threatening events, disability, and congenital malformations. The proportion of serious adverse reactions that occurred for certolizumab pegol, infliximab, golimumab, adalimumab, and etanercept was 1.79%, 1.51%, 1.17%, 1.16%, and 1.01%, respectively (Figure 1).

### The same and different points of common ADRs of five anti-TNFɑ drugs

By comparing the top 20 ADRs reported by each anti-TNFα drug in the SOCs, a total of 66 same signals were found at PTs for the five inhibitors. All common signals were sorted into Table 5. The SOC that contained the most adverse signals was infections and infestations, and the top five reports were nasopharyngitis, pneumonia, urinary tract infection, sinusitis, and lower respiratory tract infection. The second was gastrointestinal disorders, and the top five reports were nausea, diarrhea, vomiting, constipation, and Crohn’s disease.

**TABLE 5:** Same ADRs among five anti-TNFα drugs.

System organ classes	ADRs	Signal N
Blood and lymphatic system disorders	Anemia, lymphadenopathy, thrombocytopenia, leukopenia, and neutropenia	5
Cardiac disorders	Myocardial infarction, palpitations, tachycardia, and atrial fibrillation	4
Congenital, familial, and genetic disorders	Atrial septal defect and congenital anomaly	2
Ear and labyrinth disorders	Vertigo and ear pain	2
Endocrine disorders	Hypothyroidism, thyroid disorder, and adrenal insufficiency	3
Eye disorders	Visual impairment, blurred vision, and cataract	3
Gastrointestinal disorders	Nausea, diarrhea, vomiting, constipation, abdominal pain, and Crohn’s disease	6
Hepatobiliary disorders	Liver disorder, cholelithiasis, and hepatitis	3
Immune system disorders	Hypersensitivity and immune system disorder	2
Infections and infestations	Nasopharyngitis, pneumonia, urinary tract infection, sinusitis, lower respiratory tract infection, influenza, herpes zoster, and bronchitis	8
Metabolism and nutrition disorders	Decreased appetite, diabetes mellitus, dehydration, and fluid retention	4
Musculoskeletal and connective tissue disorders	Arthralgia, pain in extremity, back pain, and joint swelling	4
Benign, malignant, and unspecified neoplasms	Skin cancer, breast cancer, basal cell carcinoma, and malignant neoplasm	4
Nervous system disorders	Headache and dizziness	2
Renal and urinary disorders	Nephrolithiasis, renal failure, and dysuria	3
Respiratory, thoracic, and mediastinal disorders	Cough, dyspnea, oropharyngeal pain, and rhinorrhea	4
Skin and subcutaneous tissue disorders	Psoriasis, rash, and pruritus	3
Vascular disorders	Hypertension, hemorrhage, thrombosis, and flushing	4

When comparing the top 20 ADRs reported for each anti-TNFα drug in the SOCs, all the five TNFα inhibitors had different PTs of ADR in congenital, familial, and genetic disorders (Table 6). The number of distinctive symptoms for adalimumab, infliximab, etanercept, golimumab, and certolizumab pegol was three, six, five, four, and seven, respectively.

**TABLE 6:** Different ADRs among five anti-TNFα drugs.

System organ classes	Adalimumab	Infliximab	Etanercept	Golimumab	Certolizumab pegol
Blood and lymphatic system disorders				Myelosuppression	
Cardiac disorders	Heart valve incompetence	Acute coronary syndrome	Cardiac valve disease		Sinus tachycardia
Congenital, familial, and genetic disorders	Type IV hyperlipidemia	Gilbert’s syndrome, congenital foot malformation, and hemophilia	VACTERL syndrome	Osteogenesis imperfecta	Clinodactyly
Endocrine disorders				Acromegaly	
Eye disorders					Swelling of the eyelid and blepharospasm
Gastrointestinal disorders			Toothache		
Pregnancy, puerperium, and perinatal conditions		Placenta previa	Induced labor		Twin pregnancy and placenta previa
Metabolism and nutrition disorders	Reduced fluid intake	Hypomagnesemia	Type 1 diabetes mellitus	Hypovolemia	
Immune system disorders					Iodine allergy

## Discussion

The SRS has been utilized in pharmacovigilance for safety assessment of suspected AEs due to inherent limitations of clinical trials, such as stringent trial design, strict enrollment criteria, relatively small sample size, and limited follow-up duration. Furthermore, study data from clinical trials may not fit the real world where patients and comorbidities are heterogenous. The SRS plays a major part in signal identification (Lindquist et al., 2000). At present, research on the safety signals of most drugs mainly comes from three main databases: the EudraVigilance Data Analysis System (EVDAS), Food and Drug Administration (FDA) Adverse Event Reporting System (FAERS), and WHO-VigiBase^®^ (Vogel et al., 2020). WHO-VigiAccess was launched by the WHO in 2015 to provide public access to information in VigiBase^®^, the WHO global database of reported potential side effects of medicinal products. Data mining of the WHO-VigiAccess database would provide previously unknown drug–AE associations and some well-established clinical ones (Yamoah et al., 2022). The present study was conducted to assess the post-market adverse events associated with TNFɑ inhibitors in the WHO-VigiAccess database.

Data from WHO-VigiAccess show that 70% of AEs associated with the five TNFα inhibitors are reported from the Americas, followed by Europe. Poor reporting of AEs from the African and Oceania continents had been commonly observed in other studies (Alawadhi et al., 2012; Gidudu et al., 2020). In South Africa, the lack of medical knowledge of biologic medicines among health professionals, coupled with high costs and complex procurement processes, increased barriers to the use of these drugs (Hajjaj-Hassouni et al., 2012; Martelli et al., 2017; Kvamme et al., 2020). Although the use of TNFα inhibitors to treat rheumatoid arthritis is more common among the South African doctors surveyed, accounting for about half of the prescriptions, it can be seen from this study that the number of reported adverse events in Africa is still very low (Mocke-Richter et al., 2021). This may be due to poor quality social mobilization, inadequate accessibility of adverse reaction reporting systems, and low information system coverage.

AEs were more commonly reported in females than males. Except for infliximab, the 45–64 age group had the most adverse reactions after anti-TNFα treatment. The reason for this finding is that the inhibitors of TNFα, such as infliximab, etanercept, and adalimumab, are now second line to methotrexate in RA (Papadopoulos et al., 2019). Golimumab and certolizumab pegol can be combined with methotrexate in the treatment of moderate-to-severe active RA with poor efficacy of disease-modifying anti-rheumatic drugs (DMARDs, including methotrexate) (Smolen et al., 2023). Real-world surveys based on the FAERS database found that more than half of anti-TNF therapy was used to treat rheumatoid arthritis. RA affects at least twice as many women as men, and although it can occur at any age, the peak incidence is at the age of 50 years (Deepak et al., 2013).

An AE with a reporting rate of ≥1% is usually considered common (Chen et al., 2019). Therefore, the serious adverse events, including life-threatening events, disabling, and congenital malformations, of the five anti-TNF α drugs are not common. The most common ADRs for all five TNFα inhibitors were rash, arthralgia, rheumatoid arthritis, headache, pneumonia, psoriasis, nausea, diarrhea, and pruritus.

Both patients with RA and patients with IBD have a higher incidence and severity of infectious diseases compared to the general population. The increased risk of infections in these patients seems to be attributed mainly to immunosuppressive therapies (Calvet et al., 2021). Treatment with systemic corticosteroids is associated with a very high risk of serious infection. The risk of infection also significantly increases when anti-TNFα therapies are combined (Beaugerie and Kirchgesner, 2019; Zabana et al., 2019; Singh et al., 2020). Through a review of the English literature over the last 30 years, experts noted an increased risk of bacterial and mycobacterial infections in patients with arthritis treated with TNF inhibitors compared to non-biologic agents (Chiu and Chen, 2020).

As of November 2016, the FAERS database showed that the top five adverse reactions related to etanercept infections were nasopharyngitis, sinusitis, bronchitis, pneumonia, and influenza, accounting for 13.98%, 10.22%, 6.90%, 6.07%, and 5.54%, respectively. The top five adverse reactions of adalimumab-related infections were nasopharyngitis (15.50%), sinusitis (7.83%), pneumonia (6.23%), bronchitis (5.60%), and influenza (4.45%). The adverse reactions of infliximab-related infections were pneumonia (7.69%), *tuberculosis* (6.15%), herpes zoster (4.72%), pulmonary tuberculosis (2.53%), and sepsis (2.49%) (Chen et al., 2019). However, through the VigiAccess database, we found that the proportion of infection-related adverse reactions in etanercept was nasopharyngitis- 2.98%, sinusitis- 2.26%, pneumonia- 1.85%, lower respiratory tract infection- 1.62%, and infection- 1.60%. The proportion of infection-related adverse reactions in adalimumab was nasopharyngitis- 3.12%, pneumonia- 1.86%, infection- 1.71%, sinusitis- 1.51%, and influenza- 1.27%. Infliximab-related adverse reactions were pneumonia (3.75%), lower respiratory tract infection (1.12%), infection (1.09%), cellulitis (0.91%), and tuberculosis (0.84%). WHO-VigiAccess and FAERS, as the databases to evaluate post-marketing drug vigilance, showed differences in the types and incidence of infection-related adverse reactions caused by anti-TNFα agents. Due to the voluntary reporting of adverse events, the passive-monitoring FAERS database and WHO-VigiAccess database do not represent a complete and comprehensive count of adverse events and may lack information about reported events. In comparison, the FAERS database can display specific reports of each adverse reaction in most cases and screen eligible case reports more accurately, but professional personnel and intelligent analysis software are needed (Mikami et al., 2021). This may require WHO-VigiAccess to further provide more reporting information to the public to screen for potential links between drugs and adverse reactions in order to avoid incorrect guidance.

Another notable adverse event of anti-TNFɑ therapy is an increased risk of cancer. An initial meta-analysis showed that the treatment of RA with TNFi was associated with 3.3 times greater odds of cancer when compared to placebo (Bongartz et al., 2006). One published series described 48 cases of malignancy reported to the FDA in children on a TNF inhibitor, half of which were lymphomas (Diak et al., 2010). An analysis of TNFɑ-inhibitor-treated patients with inflammatory bowel disease (IBD) in the French National Health Insurance database also showed a higher rate of lymphoma (HR 2.41, 95% CI 1.60–3.64) compared with patients with IBD who had no TNF inhibitor exposure (Lemaitre et al., 2017). Furthermore, a study of patients with juvenile idiopathic arthritis, IBD, or psoriasis that used a Medicaid database hinted at a similar, albeit non-significant, increase in the risk of lymphoma in patients receiving TNFα inhibitor treatment (adjusted HR 2.64, 95% CI 0.93–7.51) (Beukelman et al., 2018). However, many register-based cohort studies and systematic reviews of randomized trials had not identified such an increased risk of overall cancer with the use of anti-TNFα, with the exception of an increased risk of squamous cell skin cancer risk in patients treated with abatacept (Xie et al., 2020; Chen et al., 2021; Li et al., 2021). Our results showed that the 10 most common adverse reactions reported were rash, arthralgia, rheumatoid arthritis, headache, pneumonia, psoriasis, nausea, diarrhea, pruritus, and dyspnea, and cancer-related adverse reactions were not included. Based on the definition of an AE, these may be self-limiting or temporal and are, therefore, not a cause for alarm. We also found that common cancer-related adverse reactions of five TNFα inhibitors include skin cancer, breast cancer, and basal cell carcinoma.

We found that the five TNFα inhibitors had different PTs of ADR in congenital, familial, and genetic disorders. It is worth noting that of all the biopharmaceuticals used to treat RA, only TNFα is approved for use during pregnancy and lactation. Prenatal exposure to TNFα has been shown to have no effect on T- or B-cell development (Förger et al., 2019; Kristjansdottir et al., 2019). Yet, there are some safety concerns regarding the risk of developing serious infections, such as tuberculosis*,* due to detectable anti-TNF antibodies in infants’ sera (Romanowska-Próchnicka et al., 2021). Certolizumab pegol and etanercept are approved by the European League Against Rheumatism (EULAR) for use during pregnancy and breastfeeding. Because the concentration of etanercept in breast milk is low and not detectable in neonatal serum, the European registry considers etanercept to be safe, although the level of evidence for etanercept is lower than that for certolizumab pegol (Clowse et al., 2018; Emery et al., 2020). Data on the teratogenic effects of TNFα are limited, and there is no strong evidence of potentially harmful effects if used in the preconception period (Beltagy et al., 2021).

There is increasing data on ocular adverse events associated with anti-TNFα therapy (Nicolela and Pavesio, 2020). Our study found that the most common ocular adverse reactions caused by the five TNFα inhibitors were visual impairment and blurred vision, and the swelling of the eyelid and blepharospasm caused by certolizumab pegol was prominent.

The use of a spontaneous reporting system database has some important implicit limitations because reporting is influenced by factors such as notoriety bias, selection bias, and under-reporting (Faillie, 2019). Missing data, as observed in the results of the current study where some AEs were reported, can neither be attributed to males nor females as well as age groups. In addition, since the VigiAccess database of the WHO is cumulative data, the ADRs of every year cannot be obtained. When drugs are put on the market at different times, the number of ADRs collected is quite different, and the signal difference of all target inhibitors cannot be compared at the same time. Therefore, further data mining will be not possible. In this study, the number of ADRs over the past years and the number of PTs were collected, and the rate of ADR reports of different drugs was compared to avoid the influence of the time of drug marketing. The study results were limited to the relative results of the five TNFα inhibitors.

## Conclusion

Anti-TNFα biological agents are an important part of autoimmune diseases. The study showed that WHO-VigiAccess reported more than 1 million adverse reactions to anti-TNF antibody treatment. The ADR of these drugs was mainly concentrated in infections and infestations, gastrointestinal disorders, and blood and lymphatic system disorders. The ADR symptoms of infection and gastrointestinal disorders had been confirmed again. In addition, infusion-related reactions caused by infliximab and lower respiratory tract infections caused by golimumab were very prominent. Even though most of the ADRs were minor and self-limiting, there were some serious ADRs that could lead to hospitalization and even death. Countries should also actively conduct safety studies on biological agents, such as cohort event monitoring, to determine the causal relationship between adverse reactions and drugs. These findings could also be stored in open-access repositories for the general public to enhance their knowledge of AEs associated with biotech drugs.

## Data Availability

The original contributions presented in the study are included in the article/Supplementary Material; further inquiries can be directed to the corresponding authors.
